# Association between albumin and depression: a population-based study

**DOI:** 10.1186/s12888-023-05174-0

**Published:** 2023-10-25

**Authors:** Sabah Al-Marwani, Anwar Batieha, Yousef Khader, Mohammed El-Khateeb, Hashem Jaddou, Kamel Ajlouni

**Affiliations:** 1 Department of Public Health and Community Medicine, Faculty of Medicine, Jordan University of Science and Technology, Irbid, Jordan; 2 National Center for Diabetes, Endocrinology and Genetics, The University of Jordan, P.O. Box 13165, Amman, Jordan

**Keywords:** Psychiatric disorders, Mood disorders, Depression, Serum albumin, National survey, Jordan, Mental health

## Abstract

**Introduction:**

Albumin is the most prevalent plasma protein and is involved in a variety of critical physiological processes. Low serum albumin levels have been linked to depression symptoms in people who had recent suicide attempts and those suffering from several mental diseases such as acute episodes of mania, and schizophrenia. However, there has been little investigation into the relationship between depression and serum albumin levels in community-dwelling persons. This research aimed to examine the relationship between serum albumin and depression in a population-based sample and whether it differs depending on other possible confounders.

**Methods:**

Our data were derived from a national household population study conducted in 2017 with a sample size of 3,521 Jordanians aged > 17 years old. The Patient Health Questionnaire (PHQ-9) scale, a self-administered scale, was used to screen for depression. Concentrations of serum albumin and other medical biomarkers were measured by blood tests. Using descriptive statistics for depression distribution and multivariate logistic regression analysis, the connection between albumin levels and depression was investigated.

**Results:**

The odds ratios (ORs) for depression were significantly lower in the third and fourth quartiles of serum albumin concentration compared to the first quartile (OR = 0.64 and 0.66, respectively; P values = <0.001 and <0.001, respectively). This association was statistically significant even after controlling for variables such as gender, age, marital status, education, and occupation (OR = 0.67 and 0.75, respectively, and P values = 0.001 and 0.02, respectively), as well as after further controlling for other health status variables such as nutrition, comorbidity, body mass index, somking status, and biomedical markers such as serum calcium, phosphate, and magnesium (OR = 0.58 and 0.59, respectively, and P values = <0.001 and 0.001, respectively). Moreover, the unadjusted and adjusted odds ratios in the three regression models declined linearly with rising quartiles of serum albumin (P trend = <0.001, 0.009, and 0.001, respectively).

**Conclusions:**

Our research found an inverse relationship between serum albumin and depression. Serum albumin could be a warning measure for depression. It is required for appropriate intervention measures to be implemented.

## Introduction

Depression is one of the world’s most prevalent mental illnesses [1]. According to recent estimates, almost 280 million individuals were depressed, including 23 million children and adolescents [2]. Depression has a substantial impact on how someone eats, sleeps, and works, as well as disrupting social connections, and feelings of self-esteem [3, 4], and in severe instances may lead to suicidal thoughts and attempts [5].

Serum albumin is the most prevalent protein in human blood plasma and is involved in a variety of processes [6, 7]. It is required for the preservation of pH and osmotic pressure, as well as the transfer of different steroids, hormones, and fatty acids. Furthermore, serum albumin seems to be involved in inflammatory physiology and has been discovered as a non-enzymatic antioxidant [7]. The literature supported evidence of a link between serum albumin and depression in groups of stroke survivors, HIV patients, elderly women, and chronic liver disease patients [8–11]. There is also new data linking low blood albumin levels to depression symptoms in diverse categories of psychiatric patients, including those who have schizophrenia and those who attempted suicide [12–15]. For example, one study found an inverse relationship between serum albumin and the severity of depressive symptoms in those over the age of 45 who had a recent suicide attempt [12]. However, there has been little investigation into the association between depression and serum albumin levels in community-dwelling people with a large sample size. As a result, our research sought to evaluate the association between depression and serum albumin and whether this relationship is dependent on several potential confounders. The specific hypothesis of this research is that lower serum albumin levels significantly increase the severity and probability of developing depression.

## Materials and methods

### Sampling and data settings

The current study’s data were obtained from a national household population research investigation that was carried out in 2017. The methodology’s specifics were discussed elsewhere [16]. In conclusion, the sample was chosen from the 12 governorates that represent the North, Middle, and South of the nation. A multistage sampling procedure was used to choose the participants. Systematic sampling drew households from the catchment areas of 17 different health centers. All household members aged > 17 years old were invited to participate in the study and to report to the health center the next morning after an overnight fast. The study was approved by the National Center for Diabetes, Endocrinology, and Genetics’ Ethics Committee, the Ministry of Health, and Jordan University of Science and Technology. The subjects provided informed consent. The information was kept private and only used for research reasons. All methods were carried out in accordance with relevant guidelines and regulations. This study had a sample size of 3,521.

### Data collection and laboratory analysis

Skilled interviewers administered a standardized questionnaire that had been pilot-tested. Age, gender, education level, marital status, amount of physical activity each week, smoking habits, and the most prevalent chronic illnesses were all questioned on the survey. The chronic illness score was calculated by adding the number of chronic diseases specified by the subjects, those for which they had prescriptions, and those diagnosed by the tests performed in this research. Chronic illnesses contained a wide range of conditions such as heart diseases, diabetes, hypertension, osteoarthritis, and osteoporosis. The score of this variable was classified as 0, 1, 2, 3, or more disorders.

Three blood samples were extracted and utilized for various laboratory assays with a cannula put into the antecubital vein. Within one hour of collection, samples were centrifuged and sent to the central laboratory of the National Institute for Diabetes, Endocrinology, and Genetics in Amman, Jordan, in separate labeled tubes in ice boxes. During the research period, all biochemical measures were performed by the same team of laboratory professionals using the same procedure [16]. Height, weight, waist, and hip circumferences were all measured in the same standard way to avoid any differences or biases in the measurement results due to variations in the measurement scale, and calculated by trained researchers. Utilizing a portable stadiometer, height was measured without footwear, and the measurement was recorded to the closest 1 cm. Weight was checked and measured with a precision of 100 gm by a standard weighing scale without heavy clothes or footwear. Body mass index (BMI) was calculated by dividing the weight (kg) by the height (m2). Subjects with a BMI of 30 kg/m2 or more were deemed obese [17].

The PHQ-9 patient health questionnaire scale, a self-administered scale, was used to screen for, and assess the severity of depression. The PHQ-9 score ranges from 0 to 27, with each of the nine items scoring from 0 to 3 (0 = never; 1 = many days; 2 = half the days; 3 = almost daily). Total scores of 5, 10, 15, and 20 reflect cut points for mild, moderate, moderately severe, and severe depression, respectively [18]. This tool’s reliability and validity have been examined, and the results show that it has good psychometric qualities and a high Cronbach’s alpha [19–21]. It is also regarded as a reliable depression measure for use in both clinical and non-clinical contexts [22]. The PHQ-9 is considered a short and valid tool with good psychometric properties to screen and identify MDD [20, 23–26].

Participants who scored ≥ 10 on the PHQ-9 depression scale were deemed depressed for this study, as advised by the literature [20, 23, 24]. A cut-off score of 10 or above maximized overall combined sensitivity and specificity to detect major depression in all age subgroups [24].

### Data analysis

Data analysis was performed using SPSS version 22. The binary form of depression and several participants’ characteristics were explained using the frequency distribution. Bivariate analyses were also conducted to examine the independent distribution of depression among participants’ characteristics, which are thought to be factors for depression, using the t-test and X^2^ test. Several logistic regression models were used to estimate the odds ratios and P values for testing the relationship between albumin and depression. Depression was handled as the dependent variable in the multivariable logistic regression analysis, whereas serum albumin was regarded as the independent variable. Serum albumin was evaluated in two distinct forms: as a continuous variable and as a categorical variable utilizing albumin quartiles. Health status variables and the presence of some diseases such as liver disease, kidney disease, and ulcerative colitis greatly affect the level of serum albumin. This is explained why this variable is also included and considered as covariate in the current study.

## Results

707(20.1%) individuals of the total participants were found to have moderate to severe depression. Whereas 1,227 (34.8%) were found to have mild depression, and 1,587 (45.1%) were non-depressed. There were significant differences in the proportion of individuals who reported suffering from depression according to gender, employment status, marital status, degree of education, number of chronic conditions, and smoking. (Table 1)

**Table 1:** Frequency distribution of depression by selected characteristics

Variable			Overall n (%)		Depressed n (%)		P-value
**Sex**							<0.001
Male			1,059(30.1)		139 (13.1)		
Female			2,462 (69.9)		568 (23.1)		
**Age**							0.1
18–39			1,342 (38.1)		283 (21.1)		
40–59			1,637 (46.5)		333 (20.3)		
≥60			542 (15.4)		91 (16.8)		
**Education**							<0.001
< High school			981 (27.9)		226 (23.0)		
High school			1,266 (36.0)		268 (21.2)		
> High school			1,274 (36.1)		213 (16.7)		
**Marital status**							<0.001
Single			531 (15.1)		106 (20.0)		
Married			2,698 (76.6)		515 (19.1)		
Widow/Divorce/Separate			292 (8.3)		86 (29.5)		
**Occupation**							<0.001
Unemployed			1,868 (53.0)		434 (23.2)		
Office work			774 (22.0)		143 (18.5)		
Filed work			457 (13.0)		78 (17.1)		
Retired			422 (12.0)		52 (12.3)		
**BMI (Kg/m2)**							0.21
<30			1,956 (55.6)		378 (19.3)		
≥30			1,565 (44.4)		329 (21.0)		
**Smoking**							0.001
No			3,005 (85.3)		576 (19.2)		
Yes			516 (14.7)		131 (25.4)		
**Number of chronic diseases**							0.002
0			1,783(50.6)		316 (17.7)		
1			1,164 (33.1)		259 (22.3)		
2			451 (12.8)		95 (21.1)		
≥3			123 (3.5)		37 (30.1)		
**Albumin (g/L)**			46.04 ± 3.4*		45 ± 3.5**		0.001

* (Mean ± Sd) of non-depressed subjects

** (Mean ± Sd) of depressed cases

The serum albumin average and standard deviation (SD) of all participants were 45.89(g/L) and 3.40 (g/L) respectively.

P- value for the independent t-test for the differences in serum albumin level averages between the depressed and no depressed groups was significant (0.001). (Table 1)

Albumin levels were shown to have a significant inverse association with depression (OR = 0.96, P value = 0.001), as determined by the results of the univariate logistic regression analysis. This association also held significant after controlling for sociodemographic characteristics such as age, sex, education, marital status, and occupation (OR = 0.95, P value = 0.002), as well as after further adjusting for health status factors like smoking, body mass index (BMI), number of chronic diseases, liver and renal function, minerals levels (Ca, Mg, P) and nutrition characteristics (OR = 0.95, P value = 0.003). (Table 2)

**Table 2:** Univariate and multivariate logistic regression analyses for the association between depression and albumin. Model 1 unadjusted; model 2 adjusted for age, sex, marital status, education, occupation, and smoking; model 3 adjusted for confounders in model 2 plus other health status variables such as nutrition, comorbidity, biomedical markers such as serum calcium, phosphate, and magnesium

Variable		Depression										
		Model 1				Model 2				Model 3		
		β (95% CI)		P-value		β (95% CI)		P-value		β (95% CI)		P-value
**Albumin**		0.96 (0.90–0.99)		0.001		0.952 (0.92–0.98)		0.002		0.945 (0.92–0.98)		0.003
**Quartiles of Albumin(g/L)**												
< 43.8		1				1				1		
43.8- 45.99		0.78 (0.63–0.97)		0.02		0.80 (0.63- 1.0)		0.05		0.73 (0.56–0.92)		0.01
46–48		0.64 (0.52–0.81)		<0.001		0.67 (0.53–0.86)		0.001		0.58 (0.44–0.77)		<0.001
> 48		0.66 (0.53–0.83)		<0.001		0.75 (0.59–0.96)		0.02		0.59 (0.44–0.82)		0.001
**P for trend**				<0.001				0.009				0.001

Further examination of the data using the quartile categories of serum albumin revealed that the odds ratio (OR) for having depression reduced linearly across the quartiles of serum albumin in all models; either adjusted model 1 or unadjusted models 2 and 3 (P trend = <0.001, 0.009, and 0.001 respectively). As compared to the reference group of individuals with the lowest amount of albumin, those in the second quartile had a significantly lower odds ratio for depression. (OR = 0.78, P value 0.02). whereas there are much lower odds ratios for depression in the third and fourth quartiles (OR = 0.64, and 0.66 respectively, and P values = <0.001 and <0.001 respectively) compared to the first quartile. This was because individuals in the last two quartiles had higher albumin levels. Conducting multivariable logistic regression tests, this inverse association persisted significant even after controlling background characteristics (age, sex, education, marital status, and occupation) (OR = 0.80, 0.67 and 0.75 and P values = 0.05, 0.001, and 0.02, respectively), and after further controlling for health status variables (smoking, BMI, number of chronic diseases, liver, and renal function, minerals levels, and nutrition characteristics) (OR = 0.73, 0.58 and 0.59, respectively, and P values = 0.01, <0.001, and 0.001 respectively). (Table 2)

Finally, the univariate and multivariate linear regression analysis models showed that depression severity which was expressed by the continuous variable (score of PHQ-9) was inversely related to serum albumin level. In other words, the severity of depression was found to significantly increase with decreasing in the level of the serum albumin. (Table 3)

**Table 3:** Univariate and multivariate linear regression models for the association between the severity of depression and albumin

Variable	PHQ-9			
	Model 1		Model 2	
	Coefficient	P-value	Coefficient	P-value
**Albumin(g/L)**	-0.1	0.001	-0.084	0.006

Model 1 unadjusted; model 2 adjusted for age, sex, marital status, education, occupation, smoking, comorbidity, and biomedical markers such as serum calcium, phosphate, and magnesium

## Discussion

We used a nationwide community-based household sample of > 17-year-old male and female adults to investigate whether there is an association between the presence of depression, as defined by the PHQ-9 scale, and the serum level of albumin. According to the findings of this research, decreased serum albumin levels in Jordanians are associated with an increased odds ratio of depression. This relationship stays significant even after controlling potential socioeconomic covariates such as age, gender, educational attainment, and marital status, as well as other health confounders such as body mass index, the number of chronic diseases, smoking, nutrition, and liver and kidney function tests.

The findings of earlier studies [27–30] are supported by the findings of the current study; nevertheless, it is noteworthy to note that the prior studies used different study designs and settings. A retrospective investigation that was carried out in Taiwan employed a different scale for the diagnosis of depression (Hamilton Rating Scale for Depression (HRSD)) than the one used in our research. After taking nutrition and liver function into account, the levels of serum albumin in Taiwanese individuals with severe depressive disorder were found to be considerably lower than those seen in normal controls [28]. In a different research, blood samples were taken from twenty-four major depressive patients at the baseline and the end of a 5-week antidepressant medication. This study found that total protein serum and serum albumin concentrations were significantly lower in major depression and treatment-resistant depression cases than in healthy controls [30]. Other findings of a case-control study which included sixty-one psychiatric inpatients either with or without suicide attempts showed the means of serum albumin concentration in major depression and mania cases were significantly lower than that of the controls [14].

The function of serum albumin as a protein with free-radical scavenging characteristics may help to explain, at least in part, the role that albumin has in the development of depression [31]. Albumin is widely acknowledged as an important antioxidant, and a significant portion of the overall antioxidant capabilities of serum may be ascribed to albumin [32]. According to the findings of some research, the pathogenesis of depressive illness is connected to a surplus of free radicals. This excess of free radicals results in oxidative stress, which is believed to cause oxidative damage linked to neurodegeneration and various mental illnesses, including depression [33–37]. Previous research has shown that people who suffer from serious depressive disorders have abnormally high amounts of an oxidative product in their peripheral blood and red blood cells [38], mononuclear cells [39], cerebrospinal fluid [40], and postmortem brains [41]. Furthermore, according to the findings of other investigations, manic-depressive disorder or major depressive disorder could also be accompanied by an immune response or an acute-phase protein response [42–44]. Literature also suggests that major depression is accompanied by the activation of the inflammatory response system (IRS). Some indicators of IRS are low Zn and serum albumin levels (30).

Another possible explanation of the mechanism is that albumin acts as a transporter for minerals such as calcium and fatty acids. As a result, lowering the concentration of this blood protein may result in lower serum calcium levels. Therefore, we investigated the impact of minerals such as Ca, Mg, and P in depression and included them as covariates. Remarkably, our findings remain significant even after controlling serum minerals level.

Finally, the cross-sectional methodology of this study and the use of a self-administered scale to assess depression is its sole potential limitations since it may give a “snapshot” of depression prevalence and albumin serum levels at a single moment in time. However, other types of studies, such as randomized clinical trials, might shed additional light on the correlations of interest. However, our study’s design has high external validity since it is based on a realistic scenario and serves as a necessary precursor to more controlled investigations. Furthermore, our study’s scale used for screening depression was different from those reported and used in previous studies. Therefore, our study’s findings are of great support and significant addition to the literature. Moreover, this study is among the first studies which investigated a such relationship in communtiy-dewling people. In addition to having sufficient statistical power, which was obtained from a broad nationally representative household sample of adult males and females aged > 17 years, this study benefits from a wealth of information, many covariates, and various biomedical tests that allow us to rule out the possibility of confounding.

## Conclusions

This research demonstrates a substantial inverse relationship between depression and serum albumin. This independent connection might indicate that serum albumin is a valuable screening biomarker for depression.

## Data Availability

Raw data will be available based on reasonable requests sent to National Center for Diabetes, Endocrinology, and Genetics and to our corresponding author.

## List of abbreviations

PHQ-9: Patient Health Questionnaire-9
MDD: Major depression disorder
IRS: Inflammatory Response System
